# Webtag: a new web tool providing tags/anchors for RT-PCR experiments with prokaryotes

**DOI:** 10.1186/1472-6750-7-73

**Published:** 2007-10-25

**Authors:** Fernando Lopes Pinto, Håkan Svensson, Peter Lindblad

**Affiliations:** 1 Department of Photochemistry and Molecular Science, The Ångström Laboratories, Uppsala University, Box 523, SE-75120, Uppsala, Sweden; 2 Department of Molecular Evolution, Evolutionary Biology Centre, Uppsala University, Norbyvägen 18, SE-75236, Uppsala, Sweden

## Abstract

**Background:**

Webtag is a tool providing oligonucleotide sequences (usually called tags or anchors) that are absent from a specified genome. These tags/anchors can be appended to gene specific primers for reverse transcriptase polymerase chain reaction experiments, circumventing genomic DNA contamination.

**Results:**

The use of a relational database, in conjunction with a series of scripts written in PHP and Perl, allows the user to rapidly obtain tags that are: 1) suitable for a specific organism, and 2) compatible with other oligonucleotides to be used in the experimental procedures.

**Conclusion:**

This new web tool allows scientists to easily and rapidly obtain suitable tags for RT-PCR experiments, and is available at .

## Background

In order to better understand different aspects of metabolism it is important to study the underlying transcriptional profile. A key factor to assemble such profiles is the ability to obtain good gene expression data. For that purpose, reverse transcription polymerase chain reaction (RT-PCR) [1] became the method of choice more than a decade ago [2,3].

RT-PCR allows exponential amplification of even a very low copy number mRNA. Because of its high sensitivity, the process is very vulnerable to DNA contamination.

Unfortunately, no RNA extraction method can guarantee the absolute absence of DNA in any given sample, ultimately leading to amplification, during PCR, of both cDNA and contaminating genomic DNA [4-11].

The effects of DNA contamination can be overcome using techniques like oligo d(A) selection, intron spanning primer design, DNase I treatment or restriction endonuclease digestion [7,8,11]. However, these procedures can be time consuming, expensive and contribute to RNA degradation. Moreover, in the particular case of prokaryotes, oligo d(A) selection and intron spanning primer design are not applicable solutions.

**Figure 1:**
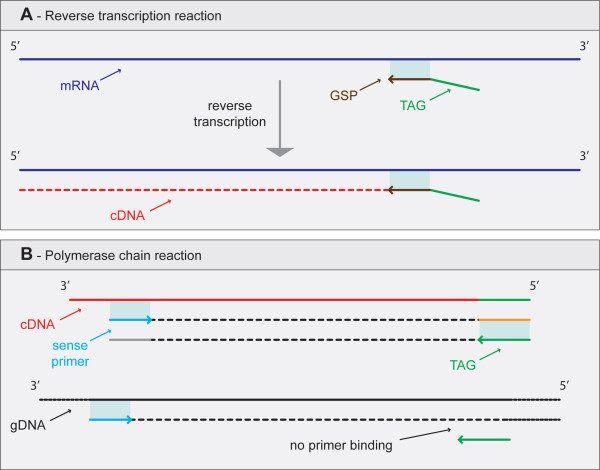
Schematic illustrating the advantages associated with the use of tags/anchors to circumvent gDNA contamination. During reverse transcriptase (A) the tag/anchor is included the cDNA allowing its specific amplification during PCR (B).

One proposed strategy leading to the specific amplification of cDNA involves the use of anchors/tags [4,9,12]. As illustrated in Figure 1, during reverse transcription of mRNA, a unique tag positioned at the 5'end of the gene specific primer is included in the cDNA. This tag will later be used as primer targeting the second cDNA strand. Application of this strategy, like in the cases of RS-PCR [4] and (EXACT) RT-PCR [9], results in RNA-specific amplification without loss of sensitivity.

Due to the constant growth in available genomic data, tags that were before considered adequate, or are even part of commercially available kits, produce significant alignments for many microorganisms when BLASTed (data not shown). Tagenerator [12] was our first effort to give the molecular biologist the possibility to associate to the potential of the above described methods with the use of genome-absent tags. The tool proved useful, but the software requirements (Perl, BioPerl [13] and BLAST [14]) and the long runtime for bigger genomes made it somewhat unpractical for some users. In order to improve the ease of use we decided to create Webtag – a web based tool that, while having the same goal as Tagenerator, would be based on improved algorithms for tag/anchor generation, much faster runtime and the possibility to handle batch runs.

## Implementation

### Basic components of the Webtag tool

The web tool consists of two components: 1) a MySQL relational database and 2) a web interface implemented in PHP, running on an Apache web server and using Perl scripts for batch processing. Prior to building the database, Perl scripts were also used in the generation of tag sequences, genome adequacy evaluation (BLAST) [14], melting temperature calculation and likelihood of secondary structure formation [15,16].

### Generation of genome adequate tags

Since specific, high yield, PCR can only be achieved using carefully designed primers, the following tag construction parameters were considered and implemented: tag length, melting temperature, GC content, absence of repeats and absence of secondary structures.

**Figure 2:**
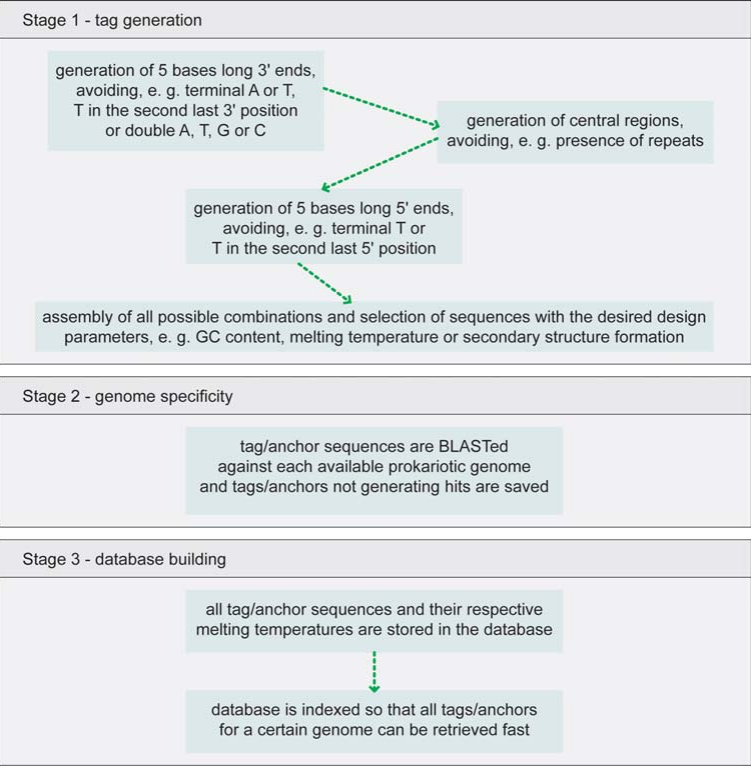
Schematic describing tag generation, tag validation and database building processes.

The tags in the Webtag relational database were constructed as shown in Figure 2. Initially, all combinations of 5 bases long sequences containing the 4 bases A, T, C and G were filtered in order to select adequate "3'end regions". Briefly, this region should contain: a) a terminal G or C (to insure specific binding), b) between 2 and 3 G's or C's (for 3'end stability) and c) never more than 2 G's or C's in a row (to avoid false priming). The generation proceeded by adding more bases to selected 3'regions. During the construction of the "central region" di-nucleotide repeats and long runs of a single base were avoided. Finally, the "5'end region" was added. Just like for the "3'end region" the length is 5 bases, but the selection parameters were more relaxed (while maintaining binding stability at the 5'end).

After full assembly, all sequences were analyzed for GC content (40% to 60%), correct melting temperature (above 52°C) [15,16] and secondary structure formation (maximum free energy must be above -4 kcal/mol for dimer formation, and above -3 kcal/mol for hairpin formation) [15]. This process resulted in the generation of more than half a million unique tags.

All tags are then tested for genome adequacy, against each prokaryotic genomic sequence found at the NCBI FTP server [17]. For this purpose BLAST settings are defined as *length *7 and *E value *10. With such settings, even statistically poor hits resulted in tag rejection.

Finally, suitable tags were then integrated in the relational database.

### Using Webtag

**Figure 3:**
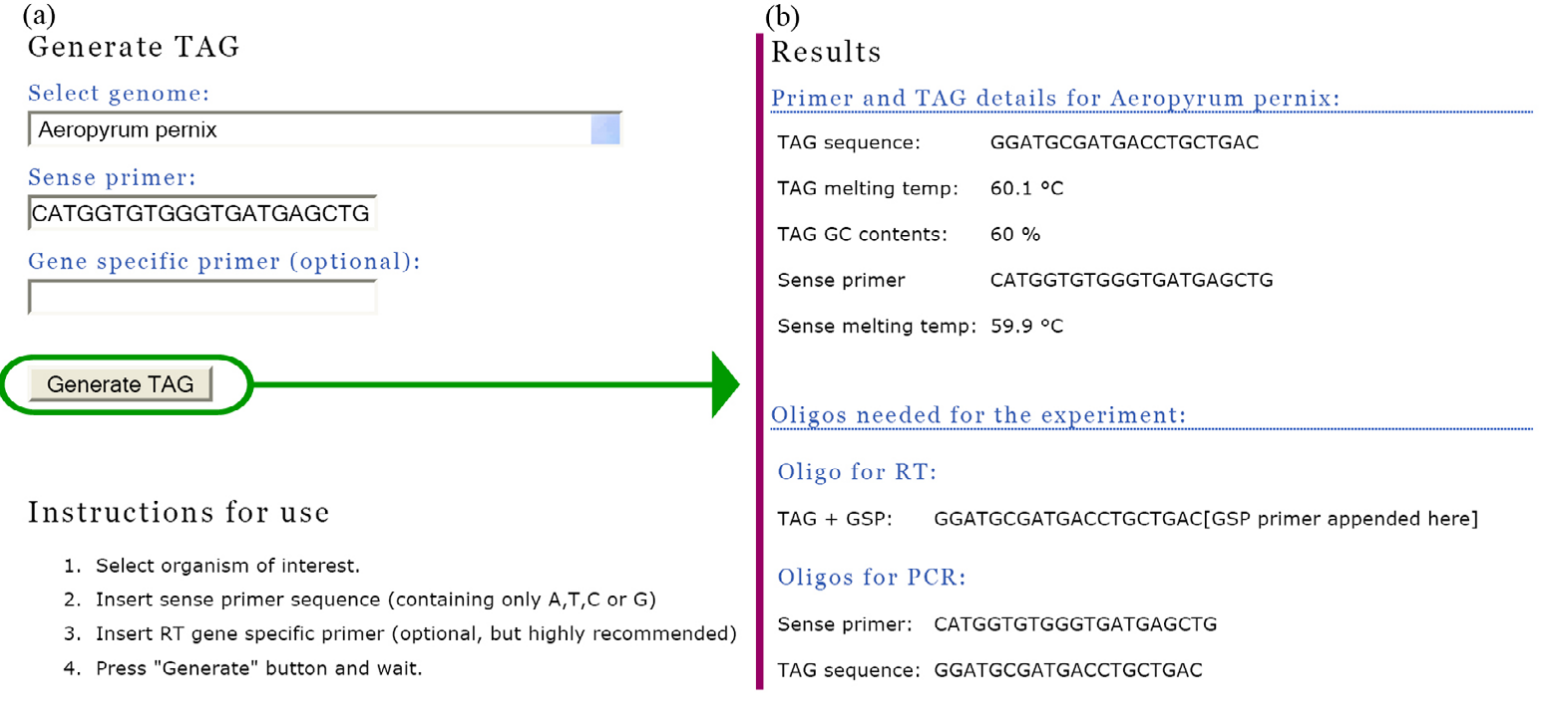
Input (a) and output (b) from the Webtag interface. After selecting a specific genome and entering the sequence of the sense primer, results (Tag sequence, melting temperatures and oligos needed for the experiment) are generated by pressing the "Generate TAG" button. For this particular example no gene specific primer was defined.

In the web interface (see Figure 3), a specific genome must be selected and a sense primer entered. Optionally, a gene specific primer may also be specified. Using the information supplied, the web interface program first calculates the melting temperature of the sense primer and then queries the database. Tags for the selected genome, having adequate melting temperatures, are extracted from the database. This output is then filtered so that the selected tag is guaranteed not to form secondary structures with either the sense primer or the gene specific primer, if one was specified.

If needed, it is possible to submit batch jobs to Webtag. When using this feature a group of sense primers must be supplied by the user (in some cases, reverse transcription gene specific primers can be specified along with the sense primers). Three types of batch jobs can be run, depending on the user's needs:

Type 1 – returns one tag for each submitted sense primer or a pair consisting of sense primer and reverse transcription gene specific primer.

Type 2 – after returning one tag for a sense primer (or a pair consisting of sense primer and reverse transcription gene specific primer), Webtag checks whether the tag may be compatible with other submitted queries. In this way, the number of needed tags is reduced.

Type 3 – only one tag will be returned, compatible with a list of sense primers submitted by the user (RT gene specific primers are not considered). The average melting temperature is calculated and used to select a set of compatible tags. These tags are then checked for secondary structure formation against all sense primers, and the first tag to have all dimerization free energies above -6 kcal/mol is output as a suitable tag.

## Results and Discussion

### Molecular biology methods

RNA extractions from *Nostoc *PCC73102 were carried-out using TRI Reagent (Molecular Research Center, Inc – USA), based on protocol given by the supplier. All RT-PCR reactions were performed in a two-step fashion using the RevertAid First Strand cDNA Synthesis Kit (Fermentas, Lithuania) and 2× PCR Master Mix (Fermentas, Lithuania), according to manufacturer's instructions. Between these two steps cDNA was cleaned using MSB Spin PCRapace kit (Invitek, Germany), following manufacturers' protocol.

### Validation of new tag generation algorithm

The new method to generate tags resulted in higher sequence variation, since it does not limit the 3' and 5' ends to a pre-defined list [12]. As a consequence, more than half a million unique sequences were found possessing good priming.

**Figure 4:**
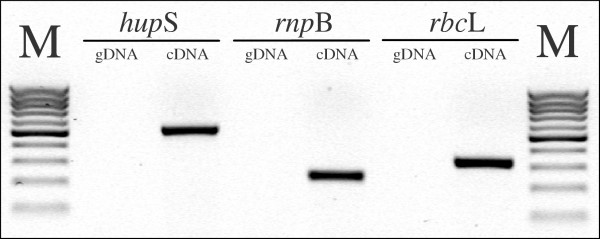
Agarose gel separation of PCR products. Lanes M – molecular weight markers (GeneRuler 100 bp DNA Ladder, Fermentas). PCR reactions using tags and sense primers (see Table 1) for three *Nostoc *PCC73102 genes – uptake hydrogenase small subunit (*hup*S), ribonuclease P subunit B (*rnp*B) and rubisco large subunit (*rbc*L). Reactions were run containing either 100 ng of genomic DNA (gDNA) or complementary DNA (cDNA) as template.

In order to test the new tag generation algorithm, 24 bases long tags were generated. This longer tag size was used to underline the possibility of performing PCR without generating products when using gDNA as template. As seen in Figure 4, using tags alongside sense primers with gDNA did not result in the synthesis of any products, while products were obtained when cDNA was the reaction template (see Table 1).

**Table 1:** Primers used in RT-PCR and generated PCR product sizes (in base pairs). During PCR the tag used as antisense primer was CAACAGACGCACGACGCAGCAGAC (**bold **in the tagged RT primer sequences).

Gene	Tagged RT primer	Sense primer	PCR product
*hup*S	**CAACAGACGCACGACGCAGCAGAC**GGTAATTCTTTAGGAACTC	CCATGTCACCCAACCCCAGCGAATCAG	533 bp
*rnp*B	**CAACAGACGCACGACGCAGCAGAC**GAAAGAGCAGTACATAAGC	AGGGTGCAAAGGTGCGGTAAGAGCG	232 bp
*rbc*L	**CAACAGACGCACGACGCAGCAGAC**GAAACGGATATCTTCTAGAC	CGTTCCGCATGACACCCCAGCC	329 bp

### Webtag database content and output

At the moment, the database holds tags suitable for 410 prokaryotic strains, downloaded from the NCBI FTP server [17]. The database will be frequently revised to include additions to NCBI.

As shown in Figure 3, the final results are returned in a simple format that facilitates primer ordering and experimental procedure planning.

## Conclusion

Webtag is a unique web service allowing the user to rapidly obtain tags that are: 1) suitable for a specific organism, and 2) compatible with other oligonucleotides to be used in the experimental procedures.

## Availability and requirements

Project name: Webtag

Project web page: 

Operating system(s): platform independent

Other requirements: web browser and internet connection

License: free for academic use

Restrictions to use by non-academics: license needed

## List of abbreviations used

BLAST – basic local alignment search tool.

cDNA – complementary deoxyribonucleic acid.

DNA – deoxyribonucleic acid.

gDNA – genomic deoxyribonucleic acid.

mRNA – messenger ribonucleic acid.

NCBI – National Center for Biotechnology Information.

PCR – polymerase chain reaction.

RNA – ribonucleic acid.

RT-PCR – reverse transcription-polymerase chain reaction.

## Competing interests

The author(s) declares that there are no competing interests.

## Authors' contributions

FLP proposed the building of Webtag. He actively participated in building Perl scripts for: generation of tag sequences, evaluation of tag quality as a primer and genome adequacy filtering. He also participated in manuscript writing and revising. Read and approved the final manuscript.

HS had the main role in the design of Webtag web service, including all PHP coding and MySQL database building and integration. He actively participated in manuscript writing and revising. Read and approved the final manuscript.

PL was the main person responsible for the establishing of strategies to test and prove the usefulness of Webtag. He actively participated in: the planning of molecular experiments, result analysis, and manuscript revising. All the funding, critical evaluation and approval for this project were his exclusive responsibility. Read and approved the final manuscript.
